# Future possibilities in migraine genetics

**DOI:** 10.1007/s10194-012-0481-2

**Published:** 2012-09-07

**Authors:** Laura Aviaja Rudkjobing, Ann-Louise Esserlind, Jes Olesen

**Affiliations:** Danish Headache Center, Glostrup Hospital, Glostrup, Denmark

**Keywords:** Genetics, Migraine, Migraine with aura, Next-generation sequencing, GWAS, Exome sequencing

## Abstract

Migraine with and without aura (MA and MO, respectively) have a strong genetic basis. Different approaches using linkage-, candidate gene- and genome-wide association studies have been explored, yielding limited results. This may indicate that the genetic component in migraine is due to rare variants; capturing these will require more detailed sequencing in order to be discovered. Next-generation sequencing (NGS) techniques such as whole exome and whole genome sequencing have been successful in finding genes in especially monogenic disorders. As the molecular genetics research progresses, the technology will follow, rendering these approaches more applicable in the search for causative migraine genes in MO and MA. To date, no studies using NGS in migraine genetics have been published. In order to gain insight into the future possibilities of migraine genetics, we have looked at NGS studies in other diseases and have interviewed three experts in the field of genetics and complex traits. The experts’ ideas suggest that the preferred NGS approach depends on the expected effect size and the frequency of the variants of interest. Family-specific variants can be found by sequencing a small number of individuals, while a large number of unrelated cases are needed to find common and rare variants. NGS is currently hampered by high cost and technical problems concurrent with analyzing large amounts of data generated, especially by whole genome sequencing. As genome-wide association chips, exome sequencing and whole genome sequencing gradually become more affordable, these approaches will be used on a larger scale. This may reveal new risk variants in migraine which may offer previously unsuspected biological insights.

## Introduction

Migraine is an episodic and disabling neurological disorder affecting roughly 14 % of the population [1]. The two most prevalent forms are migraine without aura (MO) and migraine with aura (MA) [2]. Migraine tends to run in families and has a strong genetic basis, with heritability estimates of 40–57 % [3–5]. In the rare monogenic subtype of migraine, familial hemiplegic migraine (FHM), three causative genes have been identified [6–8]. There is, however, no significant association between these genes and MO and/or MA [9]. Many linkage studies and candidate gene studies have suggested causative genes in MO and MA, but few have been replicated. Recent attempts using genome-wide association studies (GWAS) have yielded four single nucleotide polymorphisms (SNPs) that are significantly associated with migraine and recently, three additional SNPs have shown convincing association as well [10–12]. Nevertheless, only a small part of the genetic background of MA and MO has been established. This lack of success in migraine genetics depends on several factors: the heterogeneity of the migraine disease, the lack of a quantitative phenotype and the fact that not all variants associated with migraine have been discovered. There may also be rare variants which cannot be captured by the methods used so far [13, 14].

The field of molecular genetics is developing rapidly and may now have reached a point where gene finding problems can be overcome in MO and MA. The most important new methods that are relevant for MO and MA are commonly termed next-generation sequencing (NGS). Explicitly, NGS consists of whole exome sequencing (WES) and whole genome sequencing (WGS). To date, no studies using NGS methods in studies of migraine have been published.

The aim of the present study is, in the first part, to give a brief overview of the current knowledge of migraine genetics, and to introduce the new emerging molecular genetics techniques and their use in future genetic migraine research. This study does not provide a systematic review or a complete overview of the existing literature, as others have recently done this. The second part of our study is based on interviews with three genetic experts, because the genetic field is developing much faster than the literature. The experts offer their perspectives on the future of migraine genetics and we present here a summary of their main ideas.

## Background and overview of previous genetic migraine studies

### Linkage and candidate gene studies

Linkage studies are family-based and have been widely used in the search for susceptibility genes in MA and MO. This strategy is robust in identifying highly penetrant variants, such as genes of large effect in Mendelian diseases. In complex disorders caused by multiple genes, linkage studies have yielded poorer results [15]. The candidate gene approach is based on a case–control design and does not involve an analysis of large family pedigrees. Candidate gene studies depend on prior knowledge of a few selected genes, based on evidence from linkage studies or prior knowledge of the function of the gene of interest [16]. Several linkage studies have found loci with strong evidence of linkage to migraine with and without aura, and candidate gene studies have resulted in positive associations. However, most of the studies have been underpowered and for the main part, replication studies have not been done, or the replicated results have not been statistically significant (reviewed in [17, 18]). There might be several reasons for this limited success. One of the explanations is that the rare family-specific variants have a significant impact within subsets of families and these might not be replicated in other families or case–control studies. Due to this, results from different family studies will contradict each other and the susceptibility to migraine that these variants account for can be questioned [19].

### Genome-wide association studies

Genome-wide association studies have been the preferred method in the last few years. This approach is also based on a case–control design, but compared to the previous methods, GWAS is a non-hypothesis driven method. GWAS rely on the common disease-common variant model, stating that most of the genetic variation in common complex diseases is due to common variants [20, 21].

Four significant SNPs have been associated with MA and/or MO. In a recent GWAS, three additional SNPs have been convincingly associated with MO only [10–12]. The first GWAS was performed by the International Headache Genetics Consortium in a clinical-based population and identified the first SNP associated with migraine. The variant marker (rs1835740) is located on chromosome 8q22.1 between two potential candidate genes involved in glutamate homeostasis [10]. In the second migraine GWAS by Lighardt et al., different population-based cohorts were pooled together and resulted in large migraine cohorts both for the original sample and for the replication sample. Despite this, none of the SNPs investigated reached the genome-wide significant threshold [22]. In the third GWAS, three additional SNPs were found to be associated with migraine. The three variants identified were located on the chromosomes 2q37.1, 12q13.3 and 1p36.32 in regions of genes involved in glutamate homeostasis and pain mechanisms [11]. Recently, Freilinger et al. reported a GWAS where three SNPs were convincingly associated with MO. Two of the SNPs were located at 1q22 within the MEF2D gene, which regulates neuronal differentiation and restricts the number of excitatory synapses by neuronal activity-dependent activity. The third SNP was located close to the TGFBR2 gene at chromosome 3p24. This gene encodes a serine-threonine kinase, which is involved in the regulation of cell proliferation and differentiation and in the production of extracellular matrix. Two more susceptibility loci were found in the study. However, the replication of these was weak and further studies are needed [12]. All the aforementioned associations conferred only a small increase in risk, yielding odds ratios (OR) not higher than 1.36 [10–12].

Cox et al. recently did a pedigree-based GWAS in relation to migraine. They studied an isolated population of Norfolk Island with a high prevalence of migraine and found associations of SNPs within genes of the serotoninergic system. The associations might be specific for this isolated population, but might bring insights and inspiration to future research [23].

It is evident that common variants are not solely responsible for the disease phenotype, and that a proportion of the genetic predisposition will be explained by highly penetrant rare variants. These rare variants will not be captured with GWAS [13].

One of the disadvantages of GWAS is the subsequent work required to confirm that the identified SNPs are causally related to migraine and to verify that genes in the vicinity of the SNP are implicated in migraine pathogenesis. Hence, the identification of a variant does not mean that the causative gene has been found. On the contrary, the variant is often located in non-coding regions many kilobases away from a gene. Finding the causative gene may thus require tremendous work first, by fine mapping the genomic area of interest and then by performing functional studies [13, 14, 24].

### Next-generation sequencing

A GWAS using 600,000 markers can give a sufficient sample size and reveal common variants with a population frequency down to 5 %. To reveal variants with a population frequency lower than 5 %, more markers and new technologies are required. If a GWAS with 1 million markers is used in large samples combined with the imputing of un-genotyped markers, it is possible to capture most of the genetic variation down to a population frequency of 1–2 % [25]. This would allow new migraine variants to be discovered [25]. In order to find very rare variants with a population frequency <1 %, it is necessary to perform exome or WGS. These methods are commonly referred to as NGS [13]. The NGS approaches are expensive and complicated to analyze, but with the current advances in bioinformatic they are becoming feasible. To date, no migraine genetic studies using NGS methods have been published.

### Whole exome sequencing

Whole exome sequencing is an investigation of the nucleotide sequence in the protein coding regions, the exons. The exons constitute approximately 1 % of the human genome [26]. Knowledge from Mendelian disorders indicates that most of the causal mutations in monogenic diseases are found in the coding regions of the genome [27]. The coding regions may, however, also be a good source of rare mutations in complex diseases, such as migraine.

Due to the cost, early exome sequencing efforts were limited to the sequencing of a few individuals. WES has been efficient in finding de novo mutations in sporadic cases of disease by sequencing parent–child trios, in which only the offspring was affected. This approach is effective for monogenic disorders, but has also been used for complex traits such as autism, mental retardation and schizophrenia [28–30]. Although these diseases are genetically and phenotypically heterogeneous, and may be caused by mutations in several genes, knowledge of mutations with large effect in sporadic cases can be used to identify candidate genes and to provide knowledge of disease pathogenesis [28, 31].

Some of the benefits of WES are that in a single experiment nearly all the coding regions likely to contain most of the disease-causing mutations can be assayed. It is possible to identify a single or a few variants that are causal for the phenotype of interest, and to identify genes acting in pathways previously unknown. Experiences with WES from other diseases illustrate how new disease insight can be gained through this approach; some mutations causing autism are found in genes that are previously found to be involved in intellectual disability or epilepsy. This shows how one pathway may lead to different phenotypes by interaction with other factors, such as the environment [29]. Current WES methods are especially useful for identification of disease-causing variants in a single large family. There are still some remaining challenges for the usage of WES in large-scale case–control studies, in particular the high sequencing price combined with the need for big sample sizes. The latter can be exemplified by a recent exome sequencing study where a variant related to amyotrophic lateral sclerosis (ALS) was identified. The variant was found in a four-generation Italian family, in which four family members were diagnosed with ALS. Subsequent analysis demonstrated that the same variant was present in 1–2 % of a large cohort of familial ALS cases from unrelated families [32]. This study emphasizes the fact that this mutation only influences a small proportion of the disease phenotype. In order to find this rare mutation with significant reproducible results, many family members and matched controls are necessary.

The cost of WES is not the only limitation of this technique. Before the DNA is sequenced, it is cut into small fragments that facilitate the reading process. However, this results in difficulty when the origin of a sequencing fragment is to be found [33]. In addition, the current methods provide data of varying depth across the exomes, which might result in a capturing bias [34]. WES is not effective in capturing all mutation mechanisms; structural variants such as repetitive regions are likely to be missed and these might play an important role in some diseases [33]. Finally, an important limitation is that causal variants in non-coding areas are missed. To capture these, sequencing of the whole genome is necessary [26, 27].

In addition to these sequencing limitations, a challenge in relation to the use of WES in case–control studies for complex diseases will be to identify rare causal variants in unrelated cases. These variants will, due to their rarity, not be shared by all affected individuals. How to point out these variants as causes of disease remains challenging and the exact migraine sub-diagnose of each individual is of great importance [35].

Despite the limitation, WES is being used in a larger setting. A project named “The 1000 Genome Project” aims to identify nearly all variants that exist at any appreciable frequency in different populations using WES. These variants catalogued in the 1000 Genome Project can be used for the selection of SNPs to be used on next-generation GWAS chips [25].

### Whole genome sequencing

From the whole exome sequencing, the next step is WGS. This approach is costly, and complicated by massive analysis of the enormous amounts of data generated in this high-output method. Furthermore, it is currently difficult to distinguish true variants from sequencing errors. A lot of work is devoted to the development of analysis techniques that can cope with the millions of variants emerging and combine data from different variants into one analysis [36].

Whole genome sequencing has confirmed single-gene variants in families with rare diseases such as Charcot–Marie–Tooth and severe hypercholesterolemia [37, 38]. Altogether, published studies of WGS are scarce. The benefit of WGS is that the whole genome is sequenced, not just the coding regions and the regions we already know as functional. The structural variants that were likely to be missed by WES are more likely to be captured by WGS. The limitations and challenges of WGS are very much the same as mentioned for WES, as they are based on the same sequencing technology [33]. However, using current technologies, sequencing the whole genome may result in lower depth of coverage compared to exome sequencing [39]. The analysis is further complicated by a higher error rate and uncertainty about how allele frequencies of previously unknown SNPs should be estimated [36, 40]. Despite this, the most limiting factor for WGS is probably the cost. Again, as the price of this method decreases, the use of WGS may increase in the near future [41].

## The experts’ perspective

**Fig. 1:**
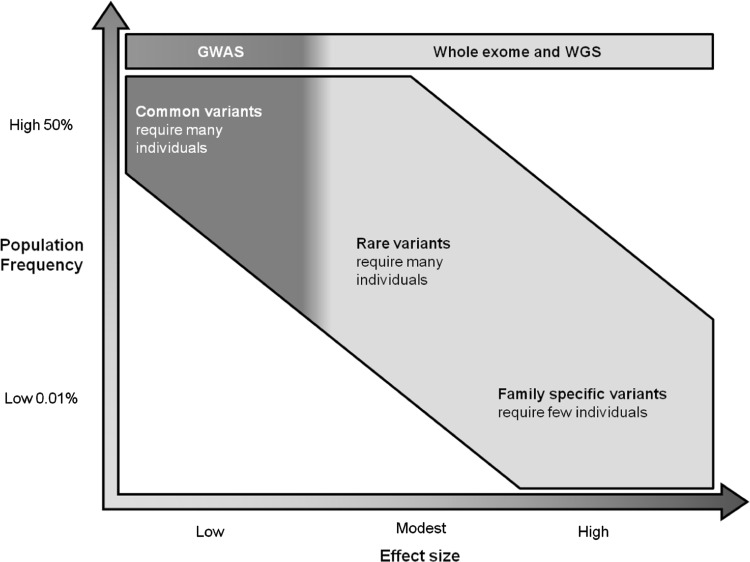
The sequencing technique of choice in relation to the effect size and the population frequency of the variants. To find common (with a population frequency down to 5 %) and rare variants (population frequency of 1–2 %) with small effect size, many individuals are required while family-specific variants can be found by sequencing few individuals. Adapted from McCarthy et al. [44]

Migraine genetic research has shown to be challenging. A mix of different techniques and approaches are required to solve the genetic questions. First, an important factor to consider when looking at migraine is the effect size of the causative variants. In familial migraine, the effect size will be high but in sporadic migraine, the effect size will be smaller. It is not clear, how big an effect size is required in order to define familial migraine. The study design depends on the effect size of the variants of interest. GWAS finds common variants (with a population frequency down to 5 %) with relatively small effect size and requires a large number of unrelated cases to provide significant results, whereas exome sequencing and WGS might be more suitable in capturing rare variants with a high effect size by sequencing a small numbers of individuals (Fig. 1).

It is possible to combine the different approaches by the imputation of WGS data into pedigrees where GWAS data are already known. This method is especially well-suited in Iceland due to its founder population characterized by long haplotypes (a set of SNPs that tend to be inherited together), and the availability of extensive genealogy information of its inhabitants in the large pedigree database “Íslendingabók”. Furthermore, in Iceland GWAS has already been completed on tens of thousands of inhabitants using a chip containing approximately 600.000 variants. Some of the genotyped Icelanders have undergone a mid-depth WGS, and a haplotype can then be created by imputing the WGS data into the chip-genotyped information. Such founder populations are ideal for the purpose of finding mutations. The discovered mutation can afterwards be investigated for a disease-causing role in more outbred populations.

**Fig. 2:**
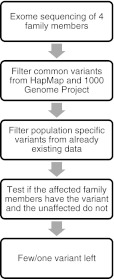
Strategy for finding variants in cases with family segregation

In regard to familial migraine molecular genetics, studies performed successfully in other complex disorders (e.g., familial diabetes or obesity) may be a source of inspiration for future migraine genetic research. In familial disease, exome sequencing has been performed in the person with the disease, the parents (where one is affected and the other parent is not) and another distantly related affected person. With this design, the mutation is assumed to be autosomal dominant. After the exomes of these four cases have been sequenced, many individual variants are expected to be found. The next step is to reduce the number of variants by filtering. The first filters applied are variants taken from the HapMap (a database cataloguing all known SNPs in the human genome [42]) or the 1000 Genome Project, since these variants are not associated with severe disease phenotypes. The next filter is population-specific exome data, because some variants occur only in certain sub-populations and are not disease-causing. After filtering, the remaining variants may be reduced to a few family-specific variants, which are thereafter tested for segregation within the whole family. The idea is that the affected family members should have the variant(s), while it should be absent in the healthy family members. In the end, this approach will yield a few or at best one variant. The process is summarized in Fig. 2. Using this design, it may suffice to examine one big family with ten or more affected individuals. These variants are likely to be family-specific and will not be found by GWAS. In cases where no strongly associated variant is found, it is assumed that the variant is not located in the coding regions, and these families become suitable candidates for WGS, when this method becomes more available.

The use of WES and WGS is still not without its limitations, such as high error rates and challenges in analyzing data. However, in the genetic research of psychiatric disorders, it has been possible to avoid the analysis problems by focusing on a specific region. One vulnerable area associated with a wide spectrum of psychiatric conditions has been identified and WGS focusing on this area only is being carried out. This strategy requires a pre-existing knowledge of a locus of interest which currently does not exist in migraine.

While the focus has been on the novel sequencing techniques, GWAS has expanded its use and possibilities with new chips containing more SNPs. This is referred to as next-generation GWAS [43]. With this approach, variants down to a population frequency of 1 % are found. Due to the large number of SNPs tested simultaneously in GWAS, the usual statistical level of 0.05 is too lenient and will result in thousands of false positive results. Therefore, the genome-wide significant level is set at 5 × 10^−8^, which is based on the testing of 1 million SNPs [44]. In rare variants, however, less than 1 % of the population carries the variant and it may therefore be necessary to test even more SNPs, requiring the genome-wide significance threshold to be lowered to at least 5 × 10^−9^. Thus, large sample sizes and collaboration between multiple research centers in consortia will be necessary.

All the mentioned approaches can be applied to migraine according to the effect size of the variants of interest. WES and WGS will be suitable in finding family-specific migraine variants, while the next-generation GWAS will be the best method in sporadic migraine cases. Currently, it is feasible to perform WES or WGS in familial migraine cases by testing if the identified variants segregate in the family. The findings should thereafter be replicated in other migraine families. Results from founder populations might give valuable insights and inspiration for future migraine genetic research. Combining all the results from common, rare and family-specific variants will greatly improve our knowledge and understanding of the migraine disease.

The NGS may explain some of the missing heritability, but part of the heritability is likely to be explained by other mechanisms such as gene–gene interactions, gene–environment interactions and epigenetics (changes in the disease or in the gene expression caused by mechanisms other than changes in the underlying DNA sequence [45]). These factors may play a role, but the mechanisms still need to be understood, which cannot be done by sequencing alone. The epigenetic factor is mentioned in a recent review by Bras et al., where speculations about the role of methylation of the genome are raised, as well as the role of gene–environment interactions. Bras et al. [33] underline the need of simultaneous study of DNA, RNA and protein to completely understand the genetic background of disease mechanisms.

## Conclusion

We have presented a brief overview of the genetic literature and interviewed genetic experts. The future of migraine genetics has a lot of potential. As genome-wide association chips, exome sequencing and WGS become more affordable, these techniques will be used on a larger scale. This may reveal new risk variants in migraine and offer new pathophysiological insights.
